# Supplementary material for: Effectiveness of dyadic interventions in improving outcomes for adults with multiple long-term conditions and/or frailty and their informal carers: A systematic review protocol
Source: PLoS One. 2026 Jan 30;21(1):e0333728. doi: 10.1371/journal.pone.0333728 (PMC12857980; doi:10.1371/journal.pone.0333728)
Supplement: Attachment — Submitted filename: PONE-D-25-50837_review feedback.docx [file pone.0333728.s002.docx]

Dear Dr. Arakelyan,

Thank you for your manuscript and the interesting topic presented. This manuscript addresses an important research area and presents a well-structured protocol. I have a few concerns regarding the methodology and focus of this protocol.

1) Methods: In the text, you claim to include articles from 2010 onward. However, in Table 1. Study selection criteria, the search inclusion is from 2016. Please clarify the inclusion criteria and remain consistent.
2) Please provide clearer examples of comparators.
3) The two-pronged aim is focusing on a) co-resident older informal caregiver and b) informal caregiver (friends, family). The value of the delineation is unclear in the manuscript and should be further elaborated.
4) The protocol has not clarified how a review of intervention effectiveness can inform the re-design, tailoring, and implementation of health services. There is a gap between effectiveness research and implementation research that the author should clearly address.
